# Impact of Watson’s human caring-based health promotion program on caregivers of individuals with schizophrenia

**DOI:** 10.1186/s12913-023-09725-9

**Published:** 2023-06-29

**Authors:** Shahpar Bagheri, Ladan Zarshenas, Mahnaz Rakhshan, Farkhondeh Sharif, Ebrahim Moghimi Sarani, Zahra Hadian Shirazi, Kathleen Sitzman

**Affiliations:** 1 Student Research Committee, Community Based Psychiatric Care Research Center, Department of Nursing, School of Nursing and Midwifery, Shiraz University of Medical Sciences, Shiraz, Iran; 2 Department of Nursing, School of Nursing and Midwifery, Shiraz University of Medical Sciences, Shiraz, Iran; 3 Department of Psychiatry, School of Medicine, Research Center for Psychiatry and Behavior Science, Ibn-E-Sina Hospital, Shiraz University of Medical Sciences, Shiraz, Iran; 4 Distinguished Watson Caring Science Scholar, East Carolina University, College of Nursing, Greenville, NC USA

**Keywords:** Caregivers, Health promotion, Schizophrenia, Sense of coherence, Watson’s Human Caring Theory, Well-being

## Abstract

**Background:**

Caring for people with schizophrenia is accompanied by challenges that impact caregiver health. We conducted this study to explore the effect of a Caring Science-Based health promotion program on the sense of coherence and well-being among caregivers of persons with schizophrenia.

**Methods:**

This randomized clinical trial with the Solomon four-group design was conducted on 72 caregivers randomly allocated into two intervention and two control groups. A health promotion program based on Watson’s theory was performed individually through five face-to-face sessions and a four-week follow-up. Settings were the psychiatric centers of the three educational, specialty, and subspecialty Ibn-e-Sina, Moharary, and Hafez hospitals affiliated with Shiraz University of Medical Sciences (SUMS), south of Iran. The data were collected using a demographic information form, the Sense of Coherence Scale, and the Caregiver Well-Being Scale. One-way ANOVA, chi-square, Kruskal–Wallis, and independent t-test were used to determine the homogeneity at baseline. In the post-test, multiple between-groups and pairwise comparisons were assessed by One-way ANOVA and Tukey’s post-hoc. Within-group comparisons were evaluated using paired t-tests. All tests were two-tailed, and the statistical level was considered 0.05.

**Results:**

Data analysis showed that the mean scores of caregiver sense of coherence and well-being from pre-intervention to post-intervention were significantly increased in the intervention groups (*p* < 0.001). At the same time, there were no significant differences in the control groups.

**Conclusion:**

The health promotion program based on Watson’s human caring theory facilitated ongoing intrapersonal, and holistic caring and improved the sense of coherence and well-being in caregivers of persons with schizophrenia. Hence, this intervention is recommended for developing healing care programs.

**Trial registration:**

https://www.irct.ir/trial/55040: IRCT20111105008011N2 (11/04/2021).

**Supplementary Information:**

The online version contains supplementary material available at 10.1186/s12913-023-09725-9.

## Background

Caregivers of persons with schizophrenia, who are often their family members, face many difficult situations during their caretaking [1]. Results of studies in Iran indicated that caregivers of persons with schizophrenia experience a broad spectrum of care pressure that can cause the caregiver’s health problems [2–8]. Caregivers’ physical and mental health is influences in the quality of home-based care. Thus, paying attention to caregivers’ health is vital in treating individuals with schizophrenia [9]. Despite suffering from various physical and mental problems related to the caregiving role, caregivers of individuals with schizophrenia often do not receive supportive healthcare services to assist them in their well-being [10]. Family caregivers of individuals with schizophrenia must be empowered to improve resilience and acceptance in caring for these individuals [11]. The findings suggest that interventions to strengthen the Sense of coherence (SOC) are necessary to support and promote the well-being of family caregivers who care for persons with schizophrenia [12, 13]. The SOC concept has been widely adopted within healthcare research. It is described as an internal resource that helps a person deal with stressful situations and feel that life is comprehensible, manageable, and meaningful [14]. There is ample evidence demonstrating relationships between the sense of coherence and well-being [12, 15, 16]. Individuals with a greater sense of coherence are more likely to respond to a stressor with adaptive strategies, thus enhancing the likelihood of a positive outcome to the situation and reducing the chance of detrimental effects on health and well-being [16]. It has been clarified that a relationship exists between SOC and caregivers' burden, health, and coping behavior. Thus, using SOC is effective for grasping the ability to care, ascertaining the support of the needs, and improving the well-being of caregivers of persons with schizophrenia [13]. Caregivers' well-being can be improved by identifying the causes of stress in family caregivers' daily lives and advocate them to cope with stress by emphasizing empowerment, capacity building, resilience, stress-reducing, and strength-based interventions [17]. A recent systematic literature review has investigated the effectiveness of psycho-educational interventions for mitigating burden and improving health outcomes of caregivers of people with schizophrenia. The findings revealed the interventions in some of those studies were helpful, and some were not. The interventions in most of these studies were performed in group training without considering the individuals’ needs and conditions and were not theory-based [18]. Many researchers point towards the possible need for a holistic approach that includes ongoing measures to support overall well-being and a healthy sense of coherence [10, 19–21]. In the present study, an intervention program based on a well-known nursing theory was utilized, which facilitated ongoing individualized, holistic, and intrapersonal caring between the participants and the researchers who worked with them.

Theoretical nursing frameworks and nursing theories can be helpful as guides for facilitating excellence in relation to nursing practice and therapeutic interventions [22]. During the past three decades, special attention has been paid to humanistic theories in nursing. One of these theories is the human caring theory, which Jean Watson introduced in 1975. Watson (1979) used the definition of health provided by the World Health Organization (WHO) and defined health as physical, mental, and social well-being and compatibility for having the highest daily living activity. Watson (1988) also described health as the harmony among mind, body, and soul. Accordingly, health refers to homogeneity between the self as perceived and the self as experienced. In other words, a lack of harmony among mind, body, and soul results in a lack of sense of coherence, eventually leading to anxiety and internal turmoil. This can be followed by disappointment, fear, and discomfort, resulting in disease if continued. According to Watson, the most effective care is performed interpersonally [23]. Philosophically, Watson’s theory aligns with the contemporary approach toward promoting individuals’ and families’ health in the community [24]. Therefore, it can be employed for taking care of patients’ caregivers [25].

Moreover, this theory is culturally compatible with nursing in different cultures, nations, and countries. In other words, it has been effectively applied in varied settings and populations [26]. Using Watson’s Human Caring Theory as a framework for professional practice supports productive, meaningful, and compassionate care in multiple dimensions of human health [27]. According to Watson, care is the core of nursing and includes interpersonal attempts to enhance and maintain health, humanity, and well-being. In this theory, health can be achieved through care based on Carative factors and Caritas processes (compassion, dignity, love, attention, and authentic presence) [26]. The Carative factors in Watson’s Human Caring Theory are as follows: 1) a humanistic-altruistic system of values, 2) installation of faith-hope, 3) cultivation of sensitivity to one’s self and to others, 4) developing a helping-trusting, human caring relationship, 5) promotion and acceptance of the expression of positive and negative feelings, 6) use of a creative problem-solving, caring process, 7) promotion of transpersonal teaching–learning, 8) provision of a supportive, protective, and/or corrective mental, physical, societal, and spiritual environment, 9) assistance with the gratification of human needs, and 10) allowance for existential-phenomenological-spiritual forces [26].

The primary aim of this study was to investigate the impact of a health promotion program based on Watson’s human caring theory on the sense of coherence among caregivers of individuals with schizophrenia. The secondary aim was to investigate its impact on caregivers’ well-being.

## Methods

### Study design

This study was a multi-arm, parallel-group randomized clinical trial with the Solomon four-group design. The Consolidated Standards of Reporting Trials statement for the study design and reporting was adopted from a previous study [28].

### Setting

This study was conducted in psychiatric centers of the three teaching, specialty, and subspecialty Ibn-e-Sina, Ostad Moharary, and Hafez hospitals affiliated with Shiraz University of Medical Sciences (SUMS). These three centers are the main psychiatric centers in southern Iran and offer inpatient and outpatient specialized psychiatric services to adults and adolescences with mental health problems.

Shiraz is the capital of Fars province, which has a population of around 1.8 million, making it the fifth most populous city in Iran and first in the southern half of the country [29].

### Participants

The participants included the family caregivers of individuals with schizophrenia who were diagnosed based on DSM-5, and their records were available at the psychiatric centers. We called and invited one of the patient's family members, who were the main caregivers and met our inclusion criteria, to participate in the study. Study enrollment started in April 2021 and ended in March 2022.

### Inclusion and exclusion criteria

The study’s inclusion criteria were 1) 18 years or older, 2) being able to speak and write in Persian, 3) taking care of a person with schizophrenia for at least a year, 4) willing to give written consent. The exclusion criteria were 1) not understanding the study methods, 2) being diagnosed with a psychiatric disorder according to DSM-5, 3) having taken part in structural interventions related to patient caring during the past six months.

### Sampling and sample size

Based on the study conducted by Sharif et al. [6], using G*Power3 software and considering the power of 90%, error of 5%, and loss rate of 25%, a 72-subject sample size was estimated for the study. The formula for the estimation of the sample size is:

$$n= \frac{{\left({sd}_{1}^{2}+ {sd}_{2}^{2}\right) \times \left({Z}_{1-\alpha /2} + {Z}_{1-\beta }\right)}^{2}}{{\left({\mu }_{1}- {\mu }_{2}\right)}^{2}}$$

Sd_1_ = 5.44 Sd_2=_ 6.5 Z_1-α/2_ = 1.96 Z_B_ = 1.28 µ_1_ = 13.03 µ_2_ = 18.34 effect size Cohen’s d = 0.88.

The participants were randomly allocated in blocks of 9 into intervention 1, intervention 2, control 1, and control 2 groups using a computerized random number generator (Winpepi software), ensuring a 1:1:1:1 allocation ratio. Each block, therefore, allocated 18 individuals to each of the four groups (two intervention groups and two control groups). The Solomon four-group design was used to eliminate the effect of the pretest on sensitizing the participants and prevent problems in the external validity of the research. This method is one of the strongest experimental designs, which neutralizes the negative effects of the pre-test [30].

A trained nurse with a Master of Science degree who was blind to the study groups was responsible for completing the questionnaires through face-to-face interviews. At first, the nurse explained the information regarding the study process and intervention program to caregivers of individuals with schizophrenia and ensured that caregivers received sufficient information and understood the nature of the study to enable them to feel less doubtful about accepting the intervention program. They gave consent voluntarily. Participants were comfortable and able to accept or reject the information presented. They were assured that they could withdraw from the study anytime without interfering with standard care. Then the participants’ informed consent forms were obtained, and their basic information, including demographic and pre-assessment data, was collected by data collection instruments. Based on the study design, the Sense of Coherence Scale (SOC) and the Caregiver Well-Being Scale (CWBS) were completed by the intervention group 1 and control group 1 but not by those in the intervention group 2 and control group 2. The information related to the post-assessment was collected from all the study groups one month after the intervention by SOC and CWBS. Post-assessment data gathered by the same researcher is based on the standard guidelines for maintaining reliability.

### Intervention

The present study provided interventions for improving the health status of the caregivers of individuals with schizophrenia based on the Carative factors of Watson’s human caring theory.

To develop the health promotion program, initially, a literature review was conducted regarding the experiences, viewpoints, tendencies, needs of caregivers of individuals with schizophrenia and the related interventions, and a list of health needs and strategies to meet that needs was created. The identified needs of caregivers of persons with schizophrenia included psychological, physical, and social needs, lack of knowledge, and the need to know about disease management. The strategies to meet the caregiver's needs were counseling, stress management, problem-solving, decision-making, time management, coping skills, self-care, communication skills, promotion of family coexistence, and management of schizophrenia disease [1, 31–35]. The list was then arranged based on the ten Carative factors of the Caritas processes contained in Watson's human caring theory. The intervention program based on Watson's human caring theory developed to create a healing environment, intrapersonal communication, mutual teaching–learning, sensitivity, kindness towards oneself and others, meeting the caregivers' needs, recognition and acceptance of feelings, identification of life crises, and exploring the meaning of in caregiving situations [23–27, 36–40]. Afterwards, the health promotion program for caregivers of people with schizophrenia was developed according to Watson’s human caring theory using holistic practical strategies. This program was evaluated and validated by the research team, nursing specialists, psychiatrists, and a Watson Caring Science expert (see Additional file 1 for a detailed description of the content of the intervention program based on Watson’s theory in our study). The content was presented to the participant in intervention groups through PowerPoint, short video clips, audio files, pamphlets, and worksheets for homework.

The first author, a nursing instructor with experience in mental health nursing, carried out the intervention. This nursing instructor was familiar with Watson’s theory and had completed an immersive four-week Massive Open Online Course [41] entitled “Caring Science, Mindful Practice: Implementing Jean Watson’s Human Caring Theory”.

The intervention was provided individually with face-to-face interviews, consultation, training, and practice for each participant through five 90–120-min sessions with 10–14-day intervals. In each intervention session, the participants were asked questions to identify their needs, inspire motivation, and create a caring teaching–learning atmosphere, which is Caring Science principles. Then, the participants received individualized education regarding personal conditions, needs, tendencies, and preferences. They were also provided with educational pamphlets, homework assignments, and worksheets to complete during the intervention sessions. The weekly exercises included mindfulness and self-awareness exercises, journaling about daily activities, applying problem-solving exercises, time management training, stress management, communication with others, kindness towards oneself and others, basic yoga asana, and self-care. At the beginning of each session, assignments were reviewed, and clarification with further teaching was provided if necessary. To ascertain the participants’ performance on their homework, online or phone contacts were also made for 30–60 min. After the intervention, phone follow-ups with the participants were continued for four weeks. In addition, the participants could contact the researcher with questions via phone or WhatsApp. If the participants needed diagnostic and therapeutic measures due to physical or mental problems, they were referred to the physician and the psychologist who collaborated with the researcher.

**Fig. 1:**
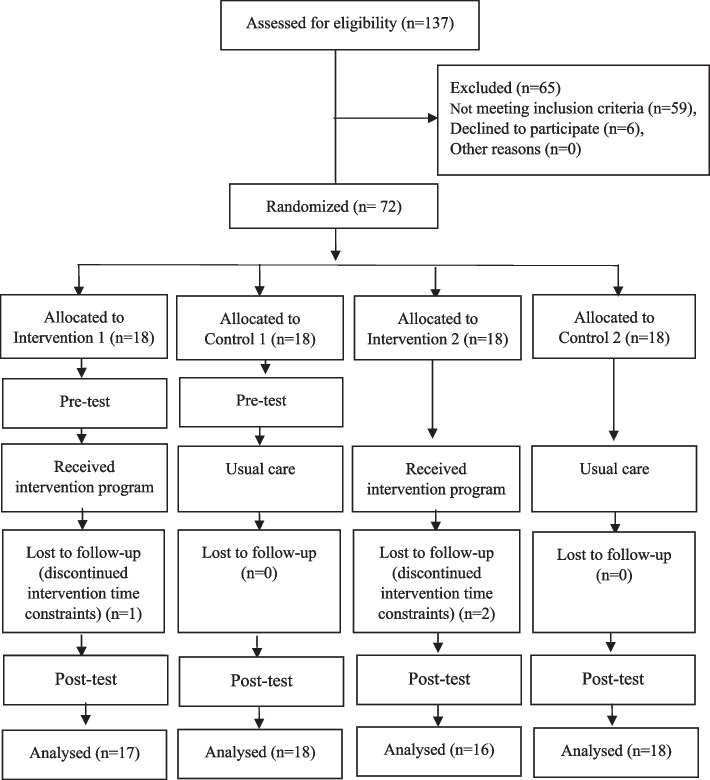
Flow chart of the study participants

The control group participants were provided with the usual care protocols, which consisted of brief oral training about schizophrenia and taking care of patients provided by a physician, psychologist, or nurse during periodical inpatient visits in the clinics. After the study, however, the control group participants were provided a consultation session by the researcher. They were also given educational content, including video clips, audio files, and pamphlets. The CONSORT flow diagram of the study is depicted in Fig. 1.

### Instruments

#### Demographic information form

This form included information about age, sex, marital status, occupation, education level, relationship with the patient, and duration of caring for the patient.

#### Caregiver well-being scale

The CWBS measures caregivers’ capabilities and compatibilities in meeting their needs and carrying out daily living activities. Tebb et al. (2013) designed a short form (14 items) of this scale that was reliable and valid. This scale has included two dimensions, i.e., well-being related to effectively managing activities of daily living and well-being concerning meeting basic needs. Participants self-reported responses to the items using a five-point Likert scale. The higher scores indicate better well-being. Cronbach’s alpha coefficients of 0.73, 0.74, and 0.83 were found for the basic needs and activities of daily living and the total scale, respectively [17]. The CWBS-14 was translated and cross-cultural adapted into Persian by Bagheri et al. [42]. The reliability and validity of the scale were approved among 144 caregivers of individuals with schizophrenia. Cronbach’s alpha coefficient was obtained as 0.84 for the whole scale. It was calculated as 0.79 for the activities of daily living dimension and 0.77 for the dimension of the basic needs.

#### Sense of coherence scale

The SOC was developed by Antonovsky (1987) [14] to measure the concept of a sense of coherence. SOC is built upon interconnected components: comprehensibility, manageability, and meaningfulness. Comprehensibility refers to the extent to which a person perceives if the world makes sense and is structured, consistent, and predictable. Manageability refers to the degree of which a person has sufficient resources to meet both external and internal demands. Finally, meaningfulness refers to the degree of which a person feels that life is a challenging but worthy emotional engagement [14]. The short form of the Sense of Coherence Scale consisted of 13 items, which could be scored from 1 to 7. Higher scores indicated a greater ability to cope with stressful situations and maintain health. The reliability and validity of this scale were evaluated by Eriksson and Lindström [43]. This scale was translated into Persian and validated by Mahammadzadeh et al. (2010). The Persian version has been tested in several Iranian populations [44, 45]; In this study, its reliability was assessed through face-to-face interviews with 30 caregivers of persons with schizophrenia. Cronbach’s alpha approved the internal consistency of the scale of 0.89.

### Data analysis

Three participants who did not attend more than one intervention session or did not do the assigned homework, were excluded from the data analysis. Descriptive statistics included frequency, percentage, mean, and Standard Deviation (SD). At first, the Kolmogorov–Smirnov test confirmed the normal distribution of the dependent variables. One-way ANOVA, chi-square, and Kruskal–Wallis tests were used to determine the homogeneity of the study groups by comparing the participants’ characteristics and scores. Also, an independent t-test was used to determine the probable differences between the participants who took the pre-test at baseline. In the post-test, One-way ANOVA was utilized to assess the differences among the four study groups regarding the mean scores of the study variables. Then, Tukey’s post-hoc test was employed for pairwise comparisons. In the groups taking part in the pre-test, changes in the outcome variable from the pre-test to the post-test were evaluated using paired t-test. All tests were two-tailed, and the statistical level was considered 0.05. All data analyses were done using the SPSS 22 software.

### Ethics

The Institutional Human Ethics Committee of Shiraz University of Medical Sciences approved this study (IR.SUMS.REC.1398.574). Then, the researchers started the sampling after trial confirmation at the Iranian Registry of Clinical Trials (IRCT) with the code IRCT20111105008011N2 (11/04/2021). Voluntary participation informed consent written forms were obtained from all the participants included in the study after providing complete information about the study objectives and procedures. Moreover, all the participants were assured about the confidentiality of their data and that they could withdraw from the study at any time without interfering with standard care. All procedures performed in this study complied with the ethical standards of the institutional and national research committee and with the 1964 Helsinki Declaration and its later amendments or comparable ethical standards.

## Results

All continuous quantitative of the study variables followed a normal distribution. Consequently, parametric tests could be employed for analyzing the data (*p* > 0.05).

Of 72 participants, 54 (75%) were female, and 18 (25%) were male. The participants’ ages ranged from 20–64 years, with a mean age of 42.59 ± 8.97 years. Caring for patients ranged from 1 to 32 years, with a mean duration of 7.70 ± 0.17. Additionally, the caregivers spent an average of 29.13 ± 9.26 h (14–42 h) taking care of patients per week. The participants’ demographic characteristics have been presented in Table 1.

**Table 1:** Demographic characteristics at the beginning of the study

Variable		Groups with pre-test *n* = 36		Groups without pre-test *n* = 36		*P*-value
		**Intervention 1** ***n*** ** = 18**	**Control 1** ***n*** ** = 18**	**Intervention 2** ***n*** ** = 18**	**Control 2** ***n*** ** = 18**	
**Sex**	Female (%)	13 (72.2)	14 (77.8)	12 (66.7)	15 (83.3)	0.68^c^
	Male (%)	5 (27.8)	4 (22.2)	6 (33.3)	3 (16.7)	
**Age (years)**	Mean (SD)	41.11 ± 8.00	45.72 ± 8.29	39.22 ± 11.23	44.33 ± 7.03	0.11^a^
**Care hours/week**	Mean (SD)	27.38 ± 11.08	28.05 ± 7.46	31.88 + 8.04	29.22 + 10.12	0.64^b^
**Marital status**	Married (%)	15 (83.3)	13 (72.2)	12 (66.7)	14 (77.8)	0.68^c^
	Single (%)	3 (16.7)	5 (27.8)	6 (33.3)	4 (22.2)	
**Education level**	Below diploma (%)	11 (61.1)	8 (44.4)	7 (38.9)	9 (50.0)	0.71^c^
	Diploma (%)	5 (27.8)	7 (38.9)	9 (50.0)	5 (27.8)	
	Academic (%)	2 (11.1)	3 (16.7)	2 (11.1)	4 (22.2)	
**Occupation**	Jobless (%)	9 (50.0)	15 (83.3)	9 (50.0)	13 (72.2)	0.09^c^
	Employed (%)	9 (50.0)	3 (16.7)	9 (50.0)	5 (27.8)	
**Care history (years)**	1–5 (%)	4 (22.2)	4 (22.2)	9 (50.0)	7 (38.9)	0.45^c^
	5–10 (%)	8 (44.4)	6 (33.3)	5 (27.8)	7 (38.9)	
	> 10 (%)	6 (33.3)	8 (44.4)	4 (22.2)	4 (22.2)	
**Relationship with patient**	Parent (%)	8 (44.4)	7 (38.9)	5 (27.8)	9 (50.0)	0.94^c^
	Spouse (%)	4 (22.2)	5 (27.8)	7 (38.9)	5 (27.8)	
	Sister/brother (%)	4 (22.2)	4 (22.2)	3 (16.7)	3 (16.7)	
	Children (%)	2 (11.1)	2 (11.1)	3 (16.7)	1 (5.6)	

The data have been expressed as Mean ± SD or n (%). Significant differences between the arms were determined by one-way ANOVA^a^, Kruskal–Wallis test^b^ and chi-square test^c^. Level of significance was set at 0.05

At the baseline comparison, the four groups’ demographic characteristics were compared. As the table depicts, no significant difference was observed among the study groups regarding these features.

Based on the results presented in Table 2, there was no significant difference between intervention group 1 and control group 1 (who participated in the pre-test) regarding the mean scores of well-being and sense of coherence (*p* > 0.05). Therefore, the two groups were similar and could be compared.

**Table 2:** Comparison of the intervention group 1 and control group 1 (taking the pre-test) regarding the mean scores of well-being and sense of coherence at baseline

Variable	Dimension	Groups with the pre-test		Range of scores	**T**	**df**	***P***
		**Intervention 1 Mean ± SD**	**Control 1 Mean ± SD**				
Caregivers Well-being	Activities	25.16 ± 4.97	26.11 ± 3.86	8–40	-0.63	34	0.52
	Basic Needs	18.16 ± 3.68	18.27 ± 2.46	6–30	-0.10	34	0.91
	Total	43.33 ± 7.96	44.38 ± 5.69	14–70	-0.45	34	0.65
Sense of Coherence	Comprehensibility	17.33 ± 5.34	18.88 ± 5.97	7–35	-0.82	34	0.41
	Manageability	13.61 ± 4.60	14.00 ± 5.19	4–28	-0.23	34	0.81
	Meaningfulness	15.66 ± 4.57	16.83 ± 4.60	4–28	-0.76	34	0.45
	Total	46.61 ± 13.03	49.72 ± 14.97	13–91	0.66	34	0.51

In the post-test, the study groups were compared, and the results have been presented in Tables 3 and 4. Accordingly, a significant difference was observed between the study groups regarding the post-test mean scores (*p* < 0.01). Additionally, post-hoc test results indicated significantly higher mean scores in the two intervention groups than in the two control groups, which showed the positive effect of the intervention on the promotion of the caregivers’ health (*p* < 0.01). However, no significant difference was detected between the two intervention groups as well as between the two control groups in this regard. This revealed that confounding factors did not influence the participants’ mean sense of coherence scores and well-being scores. The pre-test was ineffective in improving the participants’ post-test mean scores, which was one of the positive points of the Solomon four-group design.

**Table 3:** Multiple between-group comparisons of the four study groups regarding the mean scores of well-being after the intervention

**Variable (Dimension of Caregivers Well-being)**	**Group**	**Group**	**Mean difference**	**Std error**	**Tukey**	**CI 95%**		**ANOVA (between groups)**	
					**Sig**	**Lower bound**	**Upper bound**	**F**	**Sig**
**Activities**	Intervention 1	Intervention 2	1.24	1.48	0.837	-2.67	5.15	16.55	0.000*
		Control 1	7.95	1.44	0.000	4.15	11.75		
		Control 2	7.61	1.44	0.000	3.81	11.41		
	Intervention 2	Control 1	6.70	1.46	0.000	2.84	10.56		
		Control 2	6.37	1.46	0.000	2.51	10.23		
	Control 1	Control 2	-0.33	1.42	0.995	-4.07	3.41		
**Basic Needs**	Intervention 1	Intervention 2	1.07	1.01	0.713	-1.58	3.73	16.55	0.000*
		Control 1	5.60	0.98	0.000	3.01	8.18		
		Control 2	5.15	0.98	0.000	2.57	7.74		
	Intervention 2	Control 1	4.52	0.92	0.000	1.90	7.15		
		Control 2	4.08	0.99	0.001	1.45	6.71		
	Control 1	Control 2	-0.44	0.96	0.967	-2.99	2.10		
**Total**	Intervention 1	Intervention 2	2.31	2.31	0.751	-3.80	8.43	19.21	0.000*
		Control 1	13.55	2.25	0.000	7.61	19.49		
		Control 2	12.77	2.25	0.000	6.83	18.71		
	Intervention 2	Control 1	11.23	2.28	0.000	5.20	17.27		
		Control 2	10.45	2.28	0.000	4.42	16.49		
	Control 1	Control 2	-0.77	2.22	0.985	-6.63	5.07		

^*^ *p* < 0.001

**Table 4:** Multiple between-group comparisons of the four study groups regarding the mean scores of senses of coherence after the intervention

**Variable (Dimension of Sense of Coherence)**	**Group**	**Group**	**Mean difference**	**Std error**	**Tukey**	**CI 95%**		**ANOVA (between groups)**	
					**Sig**	**Lower bound**	**Upper bound**	**F**	**Sig**
**Comprehensibility**	Intervention 1	Intervention 2	1.124	1.41	0.817	-2.49	4.98	10.03	0.000*
		Control 1	5.56	1.37	0.001	1.93	9.19		
		Control 2	6.22	1.37	0.000	2.59	9.85		
	Intervention 2	Control 1	4.31	1.39	0.015	0.63	8.00		
		Control 2	4.98	1.39	0.004	1.29	8.67		
	Control 1	Control 2	0.66	1.35	0.961	-2.91	4.24		
**Manageability**	Intervention 1	Intervention 2	-0.32	1.37	0.995	-3.94	3.29	7.15	0.000*
		Control 1	4.12	1.33	0.015	0.60	7.63		
		Control 2	4.34	1.33	0.009	0.83	7.85		
	Intervention 2	Control 1	4.44	1.35	0.009	0.87	8.01		
		Control 2	4.66	1.35	0.005	1.09	8.23		
	Control 1	Control 2	0.22	1.31	0.998	-3.23	3.68		
**Meaningfulness**	Intervention 1	Intervention 2	-0.11	1.14	1.00	-3.13	2.90	16.56	0.000*
		Control 1	5.27	1.11	0.000	2.33	8.20		
		Control 2	5.77	1.11	0.000	2.83	8.70		
	Intervention 2	Control 1	5.38	1.13	0.000	2.40	8.37		
		Control 2	5.88	1.13	0.000	2.90	8.87		
	Control 1	Control 2	0.005	1.09	0.968	-2.39	3.39		
**Total**	Intervention 1	Intervention 2	0.80	3.44	0.996	-8.29	9.89	13.71	0.000*
		Control 1	14.95	3.34	0.000	6.12	23.78		
		Control 2	16.34	3.34	0.000	7.51	25.17		
	Intervention 2	Control 1	14.15	3.40	0.001	5.18	23.12		
		Control 2	15.54	3.40	0.000	5.57	24.51		
	Control 1	Control 2	1.38	3.30	0.975	-7.31	10.09		

^*^ *p* < 0.001

Table 5 is presented the results of comparing the mean scores of well-being and sense of coherence in intervention group 1 and the control group 1 (that participated in the pre-test) after the intervention. The results revealed a significant difference in the pre-test and post-test mean scores of intervention group 1, which showed a significant difference in the changes in all the variables (*p* < 0.001). Thus, the intervention effectively improved the scores of well-being and sense of coherence in the caregivers of individuals with schizophrenia. However, no significant change was observed in the mean scores of well-being and sense of coherence in the control group 1 (*p* > 0.05).

**Table 5:** Within-group comparison of the mean scores of well-being and sense of coherence in the intervention group 1 and control group 1 (with pre-test) before and after the intervention

Variable	Dimension	Group	Pre-test	Post-test	Mean difference	T	df	*P*
**Caregivers Well-being**	Activities	Intervention 1	25.35 ± 5.06	32.11 ± 3.91	-6.76	4.55	16	0.00 *
		Control 1	26.11 ± 3.86	24.16 ± 4.35	1.94	1.42	17	0.171
	Basic Needs	Intervention 1	18.23 ± 3.78	22.82 ± 2.37	-4.58	4.29	16	0.001**
		Control 1	18.27 ± 2.46	17.22 ± 2.23	1.05	1.48	17	0.156
	Total	Intervention 1	43.58 ± 8.13	54.94 ± 5.82	-11.35	4.90	16	0.00 *
		Control 1	44.38 ± 5.69	41.38 ± 6.25	3.00	1.55	17	0.138
**Sense of Coherence**	Comprehensibility	Intervention 1	17.23 ± 5.49	24.11 ± 2.75	-6.88	6.93	16	0.00 *
		Control 1	18.88 ± 5.97	18.55 ± 5.02	0.33	0.72	17	0.476
	Manageability	Intervention 1	13.41 ± 4.66	18.17 ± 3.16	-4.76	7.66	16	0.00 *
		Control 1	14.00 ± 5.19	14.05 ± 4.67	-0.55	0.13	17	0.891
	Meaningfulness	Intervention 1	15.35 ± 4.51	21.88 ± 2.17	-6.52	8.89	16	0.00 *
		Control 1	16.83 ± 4.60	16.61 ± 4.08	0.22	0.88	17	0.386
	Total	Intervention 1	46.00 ± 13.17	64.17 ± 5.60	-18.17	8.30	16	0.00 *
		Control 1	49.72 ± 14.97	49.22 ± 12.64	0.50	0.63	17	0.533

^*^ *p* < 0.001

^**^ *p* = 0.001

## Discussion

The present study findings revealed the effectiveness of the health promotion program based on Watson’s human caring theory in improving the sense of coherence and well-being amongst the caregivers of individuals with schizophrenia. To our knowledge, no study has been conducted on individual care for caregivers of persons with schizophrenia using Watson’s human caring theory. Previous studies on the impacts of various educational and supportive interventions for health promotion among caregivers of persons with schizophrenia validate the present study results. The current research findings revealed an improvement in the mean scores of sense of coherence dimensions (comprehensibility, manageability, and meaningfulness) in the participants who received the care program compared to those who did not. This might be attributed to the fact that considering the Carative factors in the Caritas processes based on Watson’s human caring theory [26, 46], caregivers and the research team presenting the program entered into a mutually caring relationship in an intentionally created healing environment infused with compassion and dignity. The intervention program based on Watson’s human caring theory helped the caregivers of persons with schizophrenia to be Self-awareness via sensitization towards themselves and self-evaluation. Sensitivity towards oneself is a prerequisite for kindness towards oneself and others and results in self-acceptance and improvement of compatibility and harmony. This helped the caregivers identify their conditions, share their emotions, identify life crises and explore the meaning of life in caregiving situations. Also, this intervention empowered the caregivers to identify and satisfy their needs and learn how to reduce their stress and manage their lives. Similarly, hybrid research performed on 34 families of patients with schizophrenia in Japan disclosed the effectiveness of a supportive environment in promoting the participants’ conditions. Understanding the meaning of life, learning how to manage the disease, or receiving support from others improved the participants’ sense of coherence [13]. Hsiao and Tsai [47] also reported that a higher sense of coherence was accompanied by increased individual and family adaptation and improved overall health for caregivers of patients with schizophrenia. Therefore, special attention is needed on therapeutic interventions that enhance these caregivers’ sense of coherence in health nursing practice.

The present study results indicated a significant difference between the intervention and control groups concerning the mean scores of the well-being of doing activities and well-being to meeting basic needs. This could result from the fact that, based on Watson’s theory, the nurses and caregivers jointly found solutions for identifying problems, meeting mental, social, and spiritual needs, solving problems associated with the loss of life routines, and fulfilling the healing process. In addition, the individuals were supported in achieving problem-solving and self-healing capabilities. Consistently, the results of controlled quasi-experimental research with pre and post-test design on caregivers of patients with schizophrenia in Iran demonstrated that training stress coping skills with an educational-mental approach effectively promoted the caregivers’ performance and well-being [48]. The results also agreed with those of the studies suggesting educational, mental, and supportive interventions to help the caregivers of persons with schizophrenia and meet their needs. These interventions included coping strategies, problem-solving, empowerment of relationships, compatibility with caretaking and life conditions [49], training in the management of schizophrenia, removal of caregivers’ health needs, emotion management training, decision-making management, social support [35], educational, mental, and social supports [1], compatibility training, training about the disease [50], time management, management of stress and role conflict [51], assistance for accepting the life conditions, making use of free time [52], and coping with complicated grief, emotional distress, and disappointment [52].

The current research’s care program intervention was based on Watson’s Human Caring theory. It included a comprehensive plan for holistic health promotion through individually tailored education and training. The program contained content related to mindfulness, attention to oneself, and self-care in physical, mental, social, and spiritual dimensions. Other dimensions of this program included problem-solving, stress-coping techniques, effective communication, disease management skills training, and kind treatment of patients and caregivers. The creation of awareness and reliable presence using mindfulness exercises and transpersonal human relationships were the critical interventions in this theory [53], which were taken into account throughout the research.

Similarly, several studies have revealed positive interventions based on Watson’s theory. For instance, Duman, Çiçek, and Baksi [37] surveyed nursing students who reported that an educational intervention based on Watson’s human caring theory improved stress coping and reduced anxiety. Tektaş and Çham [39] also disclosed that nursing care based on Watson’s human caring theory decreased anxiety, depression, and disappointment among women with a history of miscarriage and improved their health status. Arslan‐Özkan, Okumuş, and Buldukoğlu [36] showed that intervention based on Watson’s theory positively reduced tension and increased self-efficacy and compatibility amongst infertile women. Moreover, Eric et al. [38] emphasized that nursing interventions based on Watson’s theory enhanced the quality of life for patients with hypertension. Furthermore, Gonen Senturk et al. [25] stated that caregivers of patients with Alzheimer’s disease were at risk regarding physical and mental well-being, thus requiring holistic nursing care; hence, Watson’s theory was recommended to promote these caregivers’ health.

A mixed-method systematic review indicated that educational-mental interventions to address life problems stemming from caring for someone with schizophrenia should include exercises and activities for participants to practice what they have learned during the interventions [49]. In the present study, the participants were involved in activities through video clips, pamphlets, and contacts with the researcher and were required to keep records in specific worksheets. These worksheets were then discussed jointly by the researcher and the participants.

### Limitations and strengths

One of the strong points of the current study was that the intervention was based on a nursing theory. Nursing theories are essential since they provide nurses with a framework for exploring patients’ various conditions, enabling them to analyze the situations, organize their thoughts, and give the best care for their clients. Using established theories in studies and clinical settings will facilitate nursing knowledge development and prevent the separation of theory and practice. Another strength of the study was that the intervention was individualized per the participants’ interviews, needs, tendencies, and unique characteristics, keeping with the holistic focus of Watson’s Human Caring Theory [40].

A significant limitation of this study was the logistical challenge related to sampling and implementing the intervention due to the COVID-19 pandemic. All health protocols were considered as much as possible in the study process. Individual meetings helped a lot in managing this problem. The small sample size of participants may limit the generalization of our findings.

### Implication for practice

The health promotion program based on Watson’s human caring theory facilitates ongoing individualized intrapersonal caring for the holistic promotion of health and aiding individuals. Hence, this intervention program is recommended for nursing management and the development of care programs.

## Conclusion

This paper presents the findings of a study on the effectiveness of the health promotion program based on Watson’s human caring theory in improving the sense of coherence and well-being amongst caregivers of individuals with schizophrenia. This study yielded positive results and validated the efficacy of offering Caring, Science-based health promotion programs for caregivers of people with schizophrenia. It can also play a pivotal role in integrating theory with practice in psychiatric nursing.

## Supplementary Information

**Additional file 1.**

## Data Availability

The dataset used and analyzed during the current study is available from the corresponding authors upon official request.

## Abbreviations

WHO: World Health Organization
CWBS: Caregiver Well-Being Scale
SOC: Sense of Coherence Scale
