# Correction: Impact of Watson’s human caring-based health promotion program on caregivers of individuals with schizophrenia

**DOI:** 10.1186/s12913-023-09800-1

**Published:** 2023-07-13

**Authors:** Shahpar Bagheri, Ladan Zarshenas, Mahnaz Rakhshan, Farkhondeh Sharif, Ebrahim Moghimi Sarani, Zahra Hadian Shirazi, Kathleen Sitzman

**Affiliations:** 1 Student Research Committee, Community Based Psychiatric Care Research Center, Department of Nursing, School of Nursing and Midwifery, Shiraz University of Medical Sciences, Shiraz, Iran; 2 Department of Nursing, School of Nursing and Midwifery, Shiraz University of Medical Sciences, Shiraz, Iran; 3 Department of Psychiatry, School of Medicine, Research Center for Psychiatry and Behavior Science, Ibn-E-Sina Hospital, Shiraz University of Medical Sciences, Shiraz, Iran; 4 Distinguished Watson Caring Science Scholar, East Carolina University, College of Nursing, Greenville, NC, USA

**Correction to**: *BMC Health Services Research* (2023) 23:711

10.1186/s12913-023-09725-9

Following publication of the original article [1], the authors identified an error in the author name of the sixth author.

The incorrect author name is: Zahra Hadian Shiazi

The correct author name is: Zahra Hadian Shirazi

The author group has been updated above and the original article [1] has been corrected.
