# Supplementary material for: Impact of Watson’s human caring-based health promotion program on caregivers of individuals with schizophrenia
Source: BMC Health Serv Res. 2023 Jun 29;23:711. doi: 10.1186/s12913-023-09725-9 (PMC10311737; doi:10.1186/s12913-023-09725-9)
Supplement: Supplementary file 1 — Additional file 1. [file 12913_2023_9725_MOESM1_ESM.docx]

Appendix 1.

**Impact of Watson’s Human Caring-Based Health Promotion Program on Caregivers of Individuals with Schizophrenia**

**Characteristics of a Caritas nurse:** knowledge, awareness, mindfulness practice, preparedness for transpersonal communication, communication skills, practicing love and kindness towards oneself and others, sensitivity towards oneself and others through recognition, and acceptance of feelings through mindfulness exercises Caritas factors (CF) 1 and 3.

**The nurse’s preparedness in the present investigation:**The nurse, as part of a person’s environment, pays attention to care. The nurse asks herself who the caregiver is and how I can help and care for him/her (Caritas factors 1, 6, 8). To build an appropriate relationship, the nurse cautiously creates a Carative atmosphere for human relationships to occur naturally for healing and promoting the caregivers’ health by using Caritas factors 1-10.

Health Promotion Caring Program for Caregivers of Individuals with schizophrenia based on Watson’s Human Caring Theory

| 1. **First session** | **Caritas care process** | **Executive plan** |
| --- | --- | --- |
| Aim:  familiarity,  sensitivity to one’s self, Self-awareness, identification of life crises and exploring meaning of life in caregiving situation.  Length: considering the individuals’ conditions and willingness, about 1.5-2 hours (achieving goals in 1-2 sessions) | -The nurse plans to meet in a healing environment at all levels, a dignified, comfortable, quiet, private, safe, clean, and delicate environment with suitable light and temperature. The nurse uses art, beauty, and appropriate decoration, lights candles, plays light music and uses catering equipment. (For ease of access, three centers have been considered for conducting interviews in this study.  The time and location of the interviews are arranged with the caregiver (CF 1, 8, 9).  - The nurse respects the caregivers’ traditions and introduces oneself. The nurse gets close to the caregivers through friendly, warm, affectionate, and humanistic behaviors, practicing loving-kindness, compassion, empathy, patience, rapport, showing interest in forging a relationship, Sensitivity towards her/his emotions and moods, and considering them as complete individuals to facilitate her/his growth (CF 1,2, 3, 4).  - The nurse listens to the caregivers’ beliefs and appreciates her/his caregiving efforts, resulting in her/his belief in herself/himself as a valuable individual (CF 2). The nurse says, that I know that you make your best attempts to take care of your patient and your own health (CF 2, 4).  - The nurse helps the caregiver by developing a helpful and trusting relationship, considering her/his as an individual rather than a case, and creating a creative, mutual teaching-learning atmosphere. The nurse explains the sessions and says that my goal is to help you. The nurse shares one’s experiences of taking care of patients with schizophrenia and their families. The nurse says that I am there because of you, I want to be with you, and try to provide the best care and cooperate with you for solving your health problems (CF 2, 4, 6, 7, 9).  - The nurse encourages the caregiver to talk about her/his beliefs, values, stories, experiences, positive and negative emotions. The nurse helps her/him be aware of the impact of her/his experiences on her/his emotions and feelings, find the meaning of her/his life crises, and express her/his preferences and expectations from the health promotion program (CF 3, 4, 5, 10). In order to achieve these goals and enter the caregivers’ background, the nurse asks the following questions in order to promote the caregiver’s health: tell me about yourself, tell me about your life experiences, how is your condition as a caregiver of a patient who has a mental disorder, how do you perceive yourself in this condition, what does this situation mean to you, how has this experience changed your life and health status, tell me about your beliefs about spirit, do you believe in miracles, do you think that life is fair or unfair, what is your idea about suffering in life, what changes do you expect to occur in your life (CF 2, 5, 6, 9, 10).  Then, training and exercises are presented to the caregivers. The caregiver is required to do the following exercise for a week. The nurse teaches the client to be aware of her/his emotions in various situations, Sensitivity towards oneself is a prerequisite for kindness towards oneself and others and results in self-acceptance and improvement of compatibility, harmony, and health (CF 3, 7, 9, 10):  - Mindfulness practice: eating, walking, and sitting with awareness, breathing exercise, present-moment awareness.  - Practicing self-recognition and self-reflection by journaling and recording daily events: the caregivers have to write the following points in their daily notebooks: what are my problems, what are my interests, how do I feel at the moment, how do I behave towards myself, am I aware of my emotions at the moment, am I present at the moment when I am happy or sad, which causes stress in me and affects my health…. | Trainings:  -Mindfulness,  - Self-reflection,  - Journaling,  - Recording daily events |
| Assignment for the first week | - Practicing the learned points and applying them in daily life | Recording experiences and performances in worksheets |
| 1. **Second session** | **Caritas care process** | **Executive plan** |
| Aim: Identifying and satisfying mental and psychological needs.  Length: considering the individuals’ conditions and willingness, about 1.5-2 hours (achieving goals in 1-2 sessions) | Creating similar conditions to the first session provides a healing environment for continuing discussions, mutual teaching-learning, and meeting the caregiver's needs (CF 7, 8,9).  - The nurse reviews the caregiver's notes for clarification. They discuss how successful the caregiver has been in awareness through the past week. The nurse tells the caregiver to ask any questions (CF 3, 7, 10).  - For the caregiver to understand how she/he feels about her/his health and identify mental needs and problems, the nurse asks her/him the following questions and asks her/him to remember and talk about the related issues:  talk about your positive and negative feelings, what makes you sad and stressed, what makes you angry, tell me about the impact of caregiving pressure on your feelings, emotions, and mental health, have you ever felt downcast, helpless, disabled, sad, disappointed, depressed, or lonely, are you worried about continuous patient care and patient self-insufficiency, what do you do when you have these feelings, has patient caregiving affected your life, talk about the positive effects of caregiving on your health, what makes you calm and confident, what do you do to eliminate your stress, what do you do when you are angry, do you have time for a normal life, has your workload increased due to the patient's dependence on you, have your responsibilities increased, have your roles changed, talk about your health priorities, do you spend time on taking care of yourself, do you spend time on your favorite activities, what do you do, what do you like to do, tell me about your target harmony, tell me about your goals and expectations (CF 5, 6, 7, 10).  -By considering the caregivers' needs, demands, and priorities, they will achieve solutions (CF 2, 6,7, 9).  -Healing process and health promotion interventions in the internal and external healing environments will include the following: discussion about the caregivers' care pressure, expression of positive and negative feelings, stress management, understating the need for help, supporting beliefs, inducing hope, building self-confidence, training kindness towards oneself and others, problem-solving skills, time management, having daily plans, planning for the future, training decision-making skills, asking others for help or taking the patient to care centers during holidays or times the caregiver needs free time (CF 2, 7, 8, 9, 10 ).    - The caregiver is trained regarding self-healing measures for health promotion, including techniques for creating a low-stress environment, lighting candles and incense sticks, listening to music, relaxation techniques, imagination, concentration, praying, meditation, breathing exercises, art, entertainment, and daily notes (CF 1,3,8,9, 10).  If necessary, referral to a Psychologist is recommended for mental problems (9). | Training based on person’s needs:  - Stress management,  - Problem-solving,  - decision-making  - Time management |
| Assignment for the second week | Practicing and applying the learned points in daily life. | Recording experiences and performances in worksheets |
| 1. **Third session** | **Caritas care process** | **Executive plan** |
| Aim: Identifying and satisfying physical needs and problems  Length: considering the individuals’ conditions and willingness, about 1.5-2 hours (achieving goals in 1-2 sessions) | - The nurse provides similar conditions to the previous sessions, creating a healing environment for continuing discussions, eliminating ambiguities, and reviewing the caregiver's notes to clarify and answer questions (CF 3, 5, 7, 8).  - In this session, the nurse askes the following questions in a mutual teaching-learning relationship creatively to identify and satisfy the physical needs of the caregiver (CF 6, 7, 9): tell me about how you are feeling about your body, tell me about your health status, has excessive pressure consumed your energy, do you have pain in your body, do you have a headache, do you have heartburn, do you have hypertension, do you have high blood glucose and lipid levels, do you smoke cigarettes and drink alcohol, do you have a balanced diet, are you physically active, do you exercise, do you sleep sufficiently, how is your sleep quality (CF 3, 5, 6, 7, 9, 10).  By considering the caregivers' conditions, needs, demands, and priorities, they will find appropriate solutions, and the healing process will be carried out (CF 2, 6, 9, 10):  - Health promotion interventions in the internal and external healing environments will include the following:  discussion about the effects of care pressure on physical health and self-care, perceiving the importance of self-care exercises in the improvement of physical and mental health, training a balanced diet, sufficient and proper sleep, mobility, appropriate weight, avoiding high-risk behaviors, training some preliminary yoga asana and breathing exercises, teaching related to care associated with s/he physical diseases, and strengthening the caregivers in the self-healing process (1, 3, 6, 7, 9).  - If necessary, referral to a physician for physical problems (factor 9) | Trainings based on person’s needs:  - Self-care  - lifestyle promotion |
| Assignments for the third week | Practicing and applying the learned points in daily life | Recording experiences and performances in worksheets |
| 1. **Fourth session** | **Caritas care process** | **Executive plan** |
| Aim:  Identify and satisfying the problems and needs associated with family and social needs and problems  Length: considering the individuals’ conditions and willingness, about 1.5-2 hours (achieving goals in 1-2 sessions) | - Providing similar conditions to the previous sessions.  - The nurse reviews the caregiver's notes to clarification and answering questions (CF 3, 5, 7, 8).  - In this session, to identify and eliminate the problems and needs related to social and familial relationships, some questions are asked creatively and discussed in a mutual teaching-learning relationship to promote the caregivers' health (CF 3, 4, 5, 6, 7, 9).  The following questions are asked:  please talk about the impacts of caregiving on your family and social relationships, has it caused any disorders in your family members' health, do you and your family have any problems in your daily living routines and free time, talk about your family members' roles and understanding, do you have any problems in your family relationships and marital life, do you feel under pressure because of conflicts in your roles, do your family members cooperate in taking care of the patient, are you involved in social isolation,  has caregiving for the patient caused you to ignore yourself or other family members, do you have access to social supports, and have an appropriate place to live? (CF 5, 6, 7, 10).  Health promotion interventions in the internal and external healing environments based on the caregivers' needs and preferences will include the following: discussion about the impact of patient caregiving on family and social relationships, improvement of compatibility in the family, understanding communication problems, importance of listening and empathy, the importance of transparent relationships in the family, understanding the need for reducing the existing stimulants, reviewing the family expectations about the patient, emphasis on the positive aspects of family relationships, decrease of clashes and over-supporting the patient, promotion of social relationships, improvement of relationships with the family members and asking them for help, and using professional aids and the existing support resources (CF 5, 6, 7, 8, 9, 10).  If necessary, referral for family therapy and consultation with a social worker (factor 9) | Trainings based on person’s needs:  - Effective communication skills  - Life skills  - Promotion of family coexistence |
| Assignment for the fourth week | Practicing and applying the learned points in daily life | Recording experiences and performances in worksheets |
| 1. **Fifth session** | **Caritas care process** | **Executive plan** |
| Aim:  Identify and satisfying needs and problems about Disease management | The nurse is Provides similar conditions to the previous sessions.  - The nurse reviews the caregiver's notes and asks for clarification and questions (CF 3, 5, 7).  - In this session, to identify and eliminate the problems and needs related to disease management and its associated issues, the following questions are asked creatively by the nurse in a mutual teaching-learning relationship (CF 2, 3, 4, 5, 6, 7, 10): talk about your beliefs about schizophrenia, what do you know about the patient's treatments, cares, and prognosis, how have you treated the patient as well as his/her strange and violent behaviors in the face of crises, what will you do if your patient does not take medications, do you have easy access to the healthcare system and resources.  Health promotion interventions in the internal and external healing environments based on the caregivers' needs and preferences will include the following: Discussion about the problems encountered by caregivers, determination and correction of wrong beliefs about disease, training about the symptoms, causes, length, various intensities, treatments, cares, recovery, recurrence, and medications and their side effects, patient self-care, family's role in treatment and prevention of recurrence, training measures for the patient's destructive behaviors, forging relationships with the patient, decision-making for hospitalization, patient’s behavioral management, how to use emergency and home visit services, providing information about the phone numbers and addresses that can be used during crises, explanation about the treatment team, psychiatrist, psychiatric nurse, psychologist, and social worker, how to make use of the existing support services during emergencies (CF 1, 3, 4, 7, 9). | Trainings based on person’s needs:  - Management of schizophrenia disease,  - Communication with the patient and management of dangerous and strange behaviors,  - How to use educational, healthcare, and social support |
| Assignment for the fifth week | Practicing and applying the learned points in daily life | Recording experiences and performances in worksheets |
| 1. **Follow-up sessions** | **Caritas care process** | **Executive plan** |
|  | - These sessions will be held through phone contact, and time will be arranged with the caregivers in advance.  - Let the caregivers know that they have the opportunity to contact the researcher.  - Creation of a respectful, kind, and trusting atmosphere for follow-up and determining the satisfaction of needs (CF 1, 2, 3, 4, 5, 8, 9)  - The following issues will be mentioned, followed up, and resolved with the caregivers’ cooperation (CF 3, 5, 6, 7):  - Do you have any questions about the previous sessions?  - Clarifying their emotions and feelings, answering their questions (CF 5, 7)  - How successful have you been in the improvement of your body and mind harmony and promotion of your health status? (CF 7)  - What do you intend to do in the future to improve your health and harmony?  - Following up referrals | Following up and responding to the caregivers’ ambiguities and questions during the intervention and up to four weeks later |
